# First report of two successive deletions on chromosome 15q13 cytogenetic bands in a boy and girl: additional data to 15q13.3 syndrome with a report of high IQ patient

**DOI:** 10.1186/s13039-019-0432-6

**Published:** 2019-05-18

**Authors:** Maysoon Alsagob, Mustafa A. Salih, Muddathir H. A. Hamad, Yusra Al-Yafee, Jawaher Al-Zahrani, Albandary Al-Bakheet, Michael Nester, Nadia Sakati, Salma M. Wakil, Ali AlOdaib, Dilek Colak, Namik Kaya

**Affiliations:** 1 Department of Genetics, King Faisal Specialist Hospital and Research Centre, MBC: 03, Riyadh, 11211 Kingdom of Saudi Arabia; 2 Division of Pediatric Neurology, Department of Pediatrics, College of Medicine, King Saud University, Riyadh, Saudi Arabia; 3 Department of Neurosciences, King Faisal Specialist Hospital and Research Centre, Riyadh, Saudi Arabia; 4 Department of Biostatistics, Epidemiology, and Scientific Computing, King Faisal Specialist Hospital and Research Centre, Riyadh, Saudi Arabia

**Keywords:** 15q13.3 syndrome, Consecutive deletions, High IQ, *CHRNA7*, Hyperactivity, Cognitive impairment, And learning disability

## Abstract

15q13.3 syndrome is associated with a wide spectrum of neurological disorders. Among a cohort of 150 neurodevelopmental cases, we identified two patients with two close proximity interstitial hemizygous deletions on chromosome 15q13. Using high-density microarrays, we characterized these deletions and their approximate breakpoints. The second deletion in both patients overlaps in a small area containing *CHRNA7* where the gene is partially deleted. The *CHRNA7* is considered a strong candidate for the 15q13.3 deletion syndrome’s pathogenicity. Patient 1 has cognitive impairment, learning disabilities, hyperactivity and subtle dysmorphic features whereas patient 2 has mild language impairment with speech difficulty, mild dysmorphia, heart defect and interestingly a high IQ that has not been reported in 15q13.3 syndrome patients before. Our study presents first report of such two successive deletions in 15q13.3 syndrome patients and a high IQ in a 15q13.3 syndrome patient. Our study expands the breakpoints and phenotypic features related to 15q13.3 syndrome.

## Background

15q13.3 microdeletion syndrome (MS) expands on approximately 1.5 Mbp on chromosome 15q. This region is also known to involve in other diseases such as Prader Willi, Angelman syndrome, and autism [1, 2]. The syndrome has a wide spectrum of phenotypic consequences even within the same family. Patients exhibit various degrees of intellectual disabilities ranging from rarely normal intelligence to a high degree of impairment that leads to severe learning difficulties. The condition may include various dysmorphic characteristics, neurodevelopmental phenotypes, impaired vision as well as psychiatric problems, such as schizophrenia and epilepsy with recurring seizures or in some cases asymptomatic [3–8]. 15q13.3 MS may appear as de novo or could be transmitted in an autosomal dominant mode with reduced penetrance. Presence of homozygous microdeletions have also been reported [9, 10]. The prevalence of the disease remains unknown [1, 10–16]. However, a recent study estimated that 1 in 5525 live birth has a pathogenic 15q13.3 microdeletion, which can indicates that the syndrome is under-diagnosed [17].

In this study, a pool of 150 cases suspected to have chromosomal abnormalities, including dysmorphia, autism, language delay, ADHD, speech and developmental delay, were examined. The investigation was carried out by different types of oligonucleotide microarrays. Among the cohort, two cases related to 15q13.3 deletion syndrome were identified. Both patients have consecutive microdeletions separated by an intact region in the 15q13 cytogenetic bands where *CHRNA7* is partially deleted.

### Clinical presentation

*Patient 1* The patient is an 8 ½ -year-old boy presented with hyperactivity, cognitive impairment, learning disabilities and subtle dysmorphic features. He was born at term to non-consanguineous parents. Pregnancy history was not remarkable and there were no postnatal complications. He was said to be floppy initially, but managed to sit by the age of 8 months, and walked by his 15th month. Additionally, language abilities are significantly delayed and he is currently able to say comprehensible sentences of 3–4 words only. He was also noted to be hyperactive with stereotypic hands movements which improved significantly after some sessions of behavioral therapy. His parents noted improvement in his social skills after he was on methylphenidate hydrochloride with a dose of 36 mg once daily 2 years ago. His IQ was graded as 70 at the age of 6 years and was attending a special needs school. Upon examination, his weight was 21 kg at the 5th centile, height was 126.6 cm (> 25th <50th centile) and his head circumference was 53 cm (slightly above the 50th centile). He showed subtle dysmorphic features with large ears, prominent nasal tip, clinodactyly of the fifth finger and persistent fetal fingertip pads. There was one tiny pigmented naevus on the left cheek and another two (about 0.5 cm) over the abdomen and back. Cranial nerves were intact and he had normal tone, power and reflexes in the upper and the lower limbs with normal gait.

*Patient 2* The patient is a 17-year-old female who has been followed up at King Faisal Specialist Hospital and Research Center (KFSHRC) for nearly 15 years. The patient was referred to KFSHRC with the suspicion of autism and had history of speech and language delay. She had normal pre- and perinatal history with slow milestones in language areas. She was given Beery-VMI, Leiter International Performance Scale, Selected subtests, and McCarthy Scales of Children’s Abilities (MSCA) at the age of 4-year-2-month-old. She managed a 3 and 5 block figures on MSCA without difficulty for an equivalency of 4+ years. She stacked only six blocks, showed some motor clumsiness. She speaks full sentences with reasonable clarity but her vocabulary was limited. She named only one of the picture vocabulary cards and was able to point 17 out of 19. She follows directions and her receptive language skills seem relatively impact. She did extremely well on the Leiter with an age equivalency of 5-years-5-months-old and IQ of 134. On the Beery-VMI she had similar but little lesser score. She showed poor pencil control with an age of equivalency of 42 months and a standard score of 93. There were no attention related problems. She was able to toilet herself with some help but could dress and feed herself without help. She was quite irritable and demanding, particularly with her parents, but not aggressive. At the age of 6 years, she had an echocardiogram that showed a redundant mildly prolapsed mitral valve. The cardiac muscle was mildly thickened, but otherwise normal in function. At the age of 7 years she was evaluated by a neurophysiologist due to speech delay and diagnosed with expressive language delay but normal psychomotor development. Her weight was only 15.3 kg, which is far below the 5th percentile; and her height was 104.5 cm, which is also far below 5th percentile. The second ECG was also performed confirming previous findings of localized septal hypertrophy and redundant anterior mitral leaflet. At the age of 16 she has a short status found to have growth hormone deficiency and delayed puberty.

**Fig. 1:**
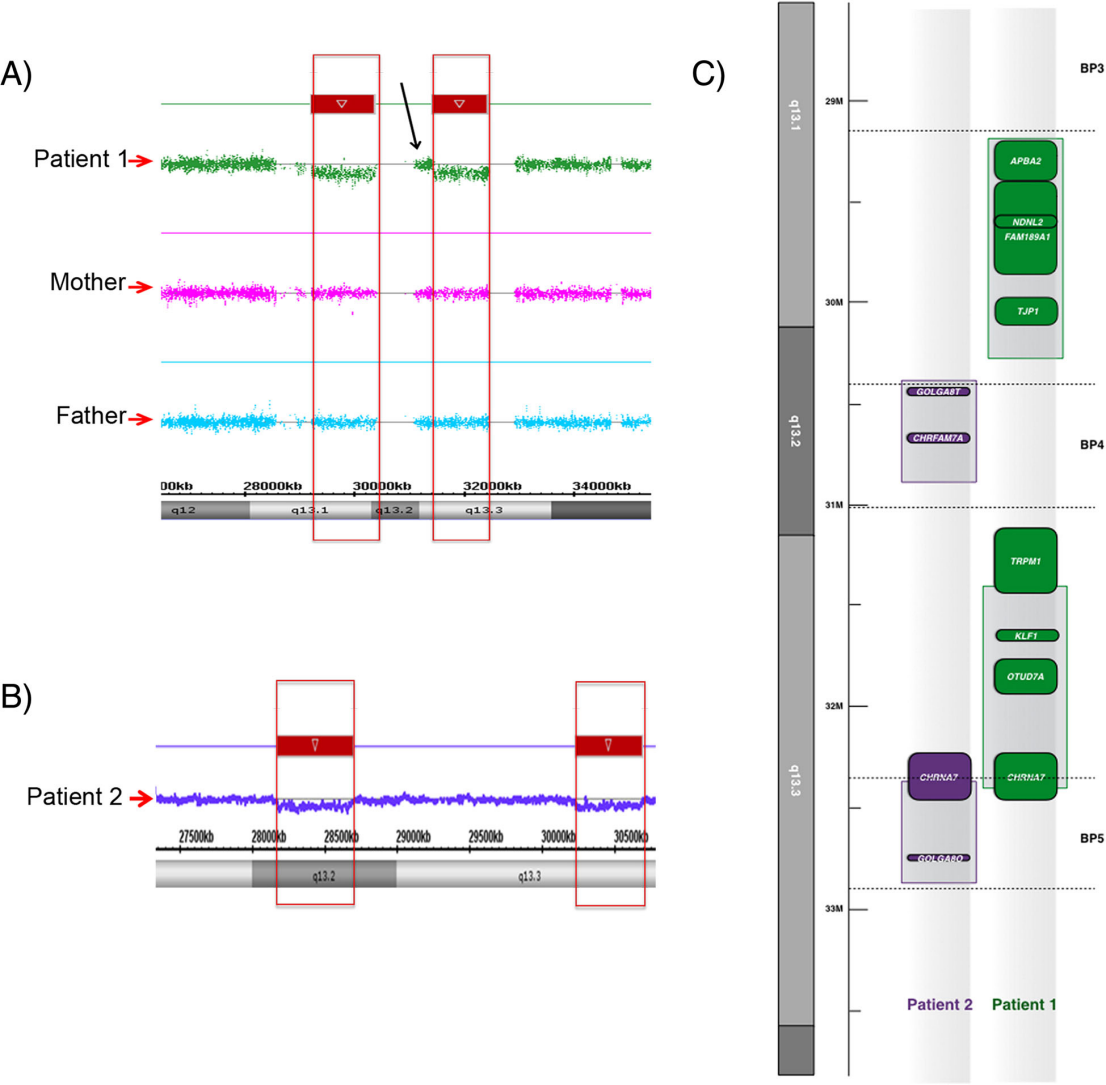
SNP array results for patient 1 and his parents, and patient 2 **a**) The patient and his parents are run on whole genome human cytogenetics 2.7 M arrays. The patient has consecutive hemizygous deletions separated by a probeless region next to a densely probed region (marked by a black arrow) comprising numerous SNP-CP probes. **b** The patient was tested on Affymetrix’s 6.0 Mapping Arrays. The analysis indicated two subsequent deletions separated by a large heavily probed region. **c** The alignment of the deletions present in both patients is shown

Molecular analysis of the patient 1 was done using high-resolution GTW-banding and high-density oligo arrays. High-resolution GTW-banding (550 band resolution) was carried out as part of diagnostic procedures and did not reveal any gross abnormality. Cytoscan HD arrays (Affymetrix Inc., San Paolo, CA, US) identified two deletions with close proximity on 15q13 cytogenetic. The second deletion sits on 15q13.3 band (Table 1, Fig. 1a). The deletion sites are covered by more than 1082 probes in the first and 1052 probes in the second deletions. There are probeless regions on both deletions making precise BP calculations difficult. Interestingly, an area covered by 435 SNP-CP probes between the deletions clearly demonstrated that these deletions are two separate unique deletions in the region (Fig. 1a). Parental DNA was tested using the same chip, no chromosomal imbalance was detected hence the hemizygous deletion in our patient is considered de novo.

**Table 1:** Summary of Molecular and Clinical Findings in Patient 1 and Patient 2 compared to other cases with 15q13.3 deletion syndrome reviewed in Lowther et al. 2015

	Patient 1	Patient 2	Lowther et al. 2015
Clinical features			
Intellectual Disabilities	Present	_	59%
High IQ	_	Present	Not reported
Autisistic Features	_	Suspected	10.9%
Speech	Delayed	Delayed	15.9%
Hyperactivity	Present	_	4.5%
Congenital Malformation	_	Present	2.4%
Dysmorphic features	Present	Present	Not indicated
Molecular Analysis			
FISH Analysis			N/A
GTW Banding			N/A
Microarrays	CytoHD	SNP Array 6.0	N/A
Double Hit			N/A
1st Deletion BP	29,215,009–30,370,019(hg19; 1155 kbp)	30,450,356–30,779,579 (hg19; 329 kbp)	N/A
2nd Deletion BP	31,444,122–32,446,830 (hg19;1003 kbp)	32,561,665–32,876,972 (hg19; 315 kbp)	N/A
1st Deletion Genes	*APBA2, NDNL2, TJP1, FAM189A1*	*GOLGA8T, CHRFAM7A*	N/A
2nd Deletion Genes	*TRPM1, KLF13, OTUD7A, CHRNA7*	*CHRNA7, GOLGA8O*	N/A

Patient 2 was also analyzed using GTW-banding that did not revealed any abnormality. However, similar to patient 1, SNP Array 6.0 (Affymetrix Inc.) indicated two consecutive deletions on 15q13. The first deletion is located on 15q13.2 while the second deletion resides on 15q13.3 (Table 1, Fig. 1b). During the analyses, we converted hg18 annotations to hg19 using UCSC’s Left Genome Annotations application for the purpose of comparison (Fig. 1c). The coordinates after the conversion are 30,450,356–30,779,579 (hg19) and 32,561,665–32,876,972 (hg19) for the first and the second deletion, respectively. The first deletion is pointed out by 95 CN and 2 SNP probes on 6.0 arrays whereas the second deletion is covered by only 43 CN probes on the same arrays. The parental DNA was not available for examination.

## Discussion and conclusions

Six break points (BPs) have been characterized in 15q region, due to the prevalence of low-copy repeats in chromosome 15q11–13 that attributes for frequent deletion and duplication in the area [2]. Among these we focus on deletions extending on 15q13.3, where 264 cases have been found in the literature [18] . Here we present two cases; a male and a female, with two separate microdeletions reside within the 15q13 cytogenetic band. Although 15q13 arm is considered one of the most instability regions of the human genome [19], to the best of our knowledge, no patient has been reported to have such pattern of deletions in this region.

Patient 1 first deletion is located in 15q13.1 cytogenetic band, small number of deletions related to PW extend to reach BP4 where the deletion resides [20]. Moreover, cases of deletions between BP3 and BP4 were reported to suggest a link between developmental and neurological disorder phenotype [21]. In addition to chromosomal rearrangement, *APBA2* (OMIM# 602712) located in the deleted area has been linked to neurological disorders. The gene encodes for amyloid precursor protein-binding protein A2. APBA2 is expressed in neuronal cytoplasm, it interacts with amyloid protein precursor and leads to the amyloid β protein production suppression, a key protein involved in Alzheimer’s pathogenesis [22]. Furthermore, duplications of *APBA2* have been associated with Schizophrenia and Autism spectrum disorders [23, 24]. The second deletion contains *OTUD7A* (OMIM# 612024) and *CHRNA7* (OMIM# 118511); both are closely associated with 15q13.3 phenotype [25]. On the other hand, cases with *CHRNA7* haploinsufficiency only manifests the 15q13.3 deletion syndrome [12, 13]. *CHRNA7* encodes α7 subunit of neuronal nicotinic acetylcholine receptor protein, among several, it is expressed the pre and post-synaptic region where it regulates both GABA and glutamate neurotransmitters in the hippocampus, it was also detected in the rat striatum dopaminergic neurons. CHRNA7 involves in Ca^2+^regulation, which then affect several Ca^2+^ dependent pathways [26]. It is worth to note that *CHRNA7* is partially deleted in our patient. This gene has 10 exons, only half of which are deleted in our case. Although *CHRNA7* is a strong candidate for the disease pathogenicity, a recent comprehensive study of *OTUD7A* human and mouse KO model, indicates that this gene contributes in brain development, moreover, abnormal morphology have been recorded in cortical neuronal and dendritic spine in mouse KO model. Hence, *OTUD7A* is a critical gene in the 15q13.3 disease manifestation and phenotypic variability [27].

The second patient’s microdeletions reside within 15q13.2 and 15q13.3. *CHRFAM7A* (OMIM # 609756) is one of the genes located within the first deletion; it is a fusion gene between a partial duplication of *CHRNA7* and *FAM7A* [28], it has a dominant negative affect in the CHRNA7 function [29], variations in *CHRFAM7A* are linked to epilepsy, schizophrenia and bipolar disorders [30, 31]. While chromosomal rearrangements and duplication in 15q13.2 have been linked to autism [32, 33], similar to patient 1, *CHRNA7* is partially deleted in the second deletion, where exons (5–10) are deleted.

Since describing 15q13.3 deletion syndrome by Sharp et al. in 2008, several reports has followed describing patients with complex variable phenotypes, included autism, seizure, dysmorphia among several, and different deletion sizes, reviewed in [18, 34]. An in depth study regarding 15q13.3 microdeletion, neuropsychiatric observations were reported in 80.5% of cases whereas developmental disabilities including speech and intellectual impairment as well as epilepsy and seizures were present in 73.6 and 28.0% of the cases, respectively. However, among the cohort, only 10–11% of patients had autism spectrum disorder or schizophrenia or mood disorder [18] and none had high IQ.

Here in, patient 1 has cognitive impairment, learning disabilities, hyperactivity and subtle dysmorphic features while patient 2 has dysmorphia, speech delay, congenital heart problems, delayed puberty and growth hormone deficiency. Interestingly patient 2 has been reported to have high IQ (IQ Score: 134). The prevalence of our patients’ phenotype in comparison to other cases reported in literature is illustrated in Table 1. It is worth mentioning that nonverbal reasoning IQ average is 60.1 [35]. It is estimated that15q13.3 microdeletion is 40–80% penetrant [17], this implies that the presence of individuals with high IQ is a possibility among carriers of the microdeletion whom does not exhibit the phenotype. Nevertheless, based on our intensive search of the literature, we have not encounter of such high IQ among the 15q13.3-S cases.

Moreover, a mouse model harboring a 15q13.3 hemizygous deletion was developed. The model demonstrated marked changes in neuronal excitability with epilepsy and schizophrenia [36]. In addition, several single gene KO mouse models have been developed in order to further understand the disease mechanism [27, 37, 38].

In summary, we present the first consecutive hemizygous microdeletions in chromosome 15q13 with atypical BPs, the two cases; a male and a female share a small overlapping region containing *CHRNA7*, and exhibit a phenotypic variation.

## Abbreviations

ADHD: Attention deficit hyperactivity disorder
BEERY VMI: Beery-Buktenica Developmental Test of Visual-Motor Integration
BPs: Breakpoints
CNV: Copy number variation
DGV: Database of genomic variants
ECG: Echocardiogram
GTW-banding: Giemsa banding using trypsin with Wright’s stain
HD: High density
IQ: Intelligence quotient
KACST: King Abdulaziz Center for Science and Technology
KFSHRC: King Faisal Specialist Hospital and Research
KSCDR: King Salman Center for disability research
Mbp: Megabasepair
MSCA: McCarthy Scales of Children’s Abilities
OMIM: Online Mendelian Inheritance in Ma

## Authors’ information

Michael Nester - recently retired from KFSHR.
